# Early-life gut microbiota in food allergic children and its impact on the development of allergic disease

**DOI:** 10.1186/s13052-023-01557-x

**Published:** 2023-11-09

**Authors:** Xiumei Yan, Jingbin Yan, Qiangwei Xiang, Huan Dai, Yinghui Wang, Lingjuan Fang, Kaiyu Huang, Weixi Zhang

**Affiliations:** 1 Department of Pediatrics, The Second Affiliated Hospital and Yuying Children’s Hospital of Wenzhou Medical University, NO. 109 Xueyuan Road, Wenzhou, Zhejiang Province 325000 China; 2 Key Laboratory of Structural Malformations in Children of Zhejiang Province, Wenzhou, Zhejiang Province 325000 China; 3 Department of Ultrasonography, Wenzhou Hospital of Integrated Traditional Chinese and Western Medicine, Wenzhou, 325000 China

**Keywords:** Food allergy, Gut microbiota, *Escherichia-Shigella*, *Bifidobacterium*

## Abstract

**Background:**

The prevalence of food allergies (FA) has been steadily increasing over 2 to 3 decades, showing diverse symptoms and rising severity. These long-term outcomes affect children’s growth and development, possibly linking to inflammatory bowel disease. However, the cause remains unclear. Previous studies reveal that early infancy significantly impacts FA development through gut microbiota. Yet, a consistent view on dysbiosis characteristics and its connection to future allergies is lacking. We explored how early-life gut microbiota composition relates to long-term clinical signs in children with FA through longitudinal research.

**Methods:**

We employed high-throughput 16S rDNA gene sequencing to assess gut microbiota composition in early-life FA children in southern Zhejiang. Follow-up of clinical manifestations over 2 years allowed us to analyze the impact of early-life gut microbiota dysbiosis on later outcomes.

**Results:**

While the diversity of gut microbiota in FA children remained stable, there were shifts in microbiota abundance. Abundant *Akkermansia*, *Parabacteroides*, *Blautia*, and *Escherichia-Shigella* increased, while *Bifidobacterium* and *Clostridium* decreased. After 2 years, two of ten FA children still showed symptoms. These two cases exhibited increased *Escherichia-Shigella* and reduced *Bifidobacterium* during early childhood. The other eight cases experienced symptom remission.

**Conclusions:**

Our study suggests that FA and its prognosis might not correlate with early-life gut microbiota diversity. Further experiments are needed due to the small sample size, to confirm these findings.

## Background

In our prior investigation, we determined that the reported prevalence of food allergy (FA) among children aged 3 to 6 years in Wenzhou was 12.86% [1]. This underscores a substantial occurrence of FA in this area. However, there remains an absence of research regarding the gut microbiota of FA-afflicted children in Wenzhou. The gut microbiota, which resides within the human gastrointestinal tract, plays a pivotal role in maintaining overall host well-being. By the age of 3 years, the human gut microbiota becomes established. Notably, studies [2, 3] have demonstrated that an imbalance in the gut microbiota prior to the age of 3 years is linked to an elevated likelihood of developing allergic diseases later in life. Nonetheless, there exists no consensus regarding the connection between early-life dysbiosis and the subsequent prognosis of allergic conditions. This study employs 16S rDNA gene amplicon sequencing to delve into the diversity and composition of gut microbiota among both FA-affected and healthy children in Wenzhou. Over a span of 2 years, we will track clinical manifestations to discern the influence of early-life changes in FA-related gut microbiota on clinical outcomes.

## Materials and methods

### Materials and reagents

The QIAamp® DNA stool mini kit was procured from Qiagen (Hilden, Germany). The NanoDrop 2000 spectrophotometer was sourced from Thermo Scientific (USA). The agarose gel DNA purification kit was obtained from Qiagen (Chatsworth, CA, USA). The TruSeq™ DNA sample preparation kit was acquired from Illumina Inc. (San Diego, CA, USA). The RNA iso™ Plus reagent was supplied by Takara (Dalian, China). Screw cap fecal containers were purchased from Sarstedt (Germany).

### Patients

This study included 20 children. From September 2019 to March 2020, 9 children diagnosed with FA by immunologists were recruited from the Immunology Outpatient Clinic. An additional child was enrolled from the Pediatric Gastroenterology Ward at The Second Affiliated Hospital of Wenzhou Medical University. Eligible participants were children aged 0 to 3 years with immunologist-diagnosed FA. Food hypersensitivity was defined as experiencing at least one specific symptom (such as gastrointestinal symptoms, rash, urticaria, swelling of lips/eyes, itchy eyes/nose, cough, and wheezing) coupled with sensitization to food allergens indicated by IgE antibody levels ≥0.35 kUA/L [4]. Among the 10 children, each presented distinct clinical symptoms of FA (refer to Table 1). Allergic symptoms were mitigated upon avoidance of corresponding allergenic foods or transition to a completely amino acid-free formula. An additional 10 non-food allergic children of matching age and gender were recruited from the local community. All FA-affected children adhered to allergen avoidance or used an entirely amino acid-free formula. After a two-year interval, we reevaluated the clinical manifestations of 10 FA-diagnosed children. This study was carried out following the principles of the Declaration of Helsinki (2013 revision). Ethical approval was granted by the Institutional Review Board at The Second Affiliated Hospital of Wenzhou Medical University, and parental consent was obtained for all participating children.

**Table 1:** Clinical characteristics of the allergic diseases in 10 children

Patient	Sex	Age(M)	Symptom	Total Ig E (IU/ml)	Specific Ig E (k UA/L)	Eosinophils
1	F	17	cough	589.2	milk 6.37	0.507 × 10^9^
2	M	31	atopic eczema, angioneurotic edema	246.7	milk 23.2, egg 6.3	0.467 × 10^9^
3	F	13	Diarrhea, atopic eczema	674.3	milk 31.1, egg 1.27	0.063 × 10^9^
4	M	14	Diarrhea, atopic eczema	794.2	milk 42.3	1.034 × 10^9^
5	M	3	Vomit, atopic eczema	452.3	milk 8.7, egg 5.7	0.965 × 10^9^
6	M	5	diarrhea	872.3	milk 7.34	0.765 × 10^9^
7	M	14	atopic eczema, dysphoria	431.5	milk 16.7, egg 64.3, crab 55.5, shrimp 61.3, soybean 9.06	0.27 × 10^9^
8	M	9	atopic eczema	342.4	milk 18.9, egg 22.3	0.578 × 10^9^
9	M	14	atopic eczema, diarrhea Hypoproteinemia, anemia	970.5	milk 6.92, egg 70.8, shrimp 28.8, crab 33.3, wheat 14.1, peanut 5.38, soybean 6.75	1.882 × 10^9^
10	F	25	atopic eczema, dysphoria	960.7	egg 15.6, milk 4.30, nuts > 100	0.364 × 10^9^

### Fecal sample collection

Parents or guardians of the 20 participants received a screw cap fecal container (Sarstedt, Germany) and a sealed plastic bag with labels. Once collected, a researcher transported the fecal samples on ice to the laboratory within 2 hours, where they were stored in − 80 °C freezers. These fecal samples were obtained from both early-life FA and non-FA children.

### 16S rDNA gene amplification and sequencing

Total DNA was extracted from thawed fecal samples using the QIAamp® DNA stool mini kit according to the manufacturer’s instructions. The extracted DNA was stored at − 20 °C for subsequent Illumina Miseq sequencing analysis. The V3-V4 region of the 16S rDNA genes was amplified from diluted DNA extracts using primers 341F (5′-CCTAYGGGRBGCASCAG-3′) and 806R (5′- GGACTACNNGGGTATCTAAT-3′). The PCR products were purified using an agarose gel DNA purification kit. An amplicon library was created with the TruSeq™ DNA sample preparation kit. The sequencing was performed using Illumina MiSeq sequencing (2 × 300 bp; Hangzhou Guhe Information and Technology, Zhejiang, China).

### Sequence analysis

Raw sequence data from 16S rDNA Miseq sequencing were demultiplexed and quality-filtered using quantitative insights into microbial ecology (QIIME) (version 1.17). Operational taxonomic units (OTUs) were clustered at a 97% similarity cutoff using UPARSE (version 7.1) and chimeric sequences were identified and removed using UCHIME [5]. The phylogenetic affiliation of each 16S rDNA gene sequence was determined using the RDP Classifier (http://rdp.cme.msu.edu/) against the SILVA (SSU119, https://www.arb-silva.de) 16S rDNA database with a confidence threshold of 70% [6]. We performed α-and β-diversity calculations and taxonomic community assessment. α-diversity metrics included rarefaction analysis, number of sequences, observed OTUs, Ace and Chao richness estimators, and Shannon and Simpson diversity indices, analyzed using MOTHUR [7]. Nonmetric multidimensional scaling (NMDS) plots based on the Bray–Curtis distance metric were used for β-diversity visualization [8]. Furthermore, we compared relative genus-level abundances between the two groups, considering genera with levels exceeding 1% within total bacteria as predominant for comparison.

### Evaluating clinical manifestation of 10 children with food allergy after 2-year follow-up

After a period of 2 years, an assessment was conducted on the children with FA to ascertain the presence of cough, allergic dermatitis, and diarrhea. The grading criteria for cough were as follows [9]: Grade I for Mild cough, Grade II for Moderate cough, and Grade III for Severe cough. The evaluation of atopic dermatitis was based on the SCORAD score [10], categorizing the condition into mild (SCORAD: 0–24 scores), moderate (SCORAD: 25–50 scores), and severe (SCORAD: > 50 scores), with the highest achievable score being 103. Stool consistency was determined according to the Bristol stool classification [11], involving the following categories: 1. Very hard and lumpy stool; 2. Hard stool with clumps; 3. Stool with cracks on the surface; 4. Smooth, soft, and sausage-like stool; 5. Soft stool with distinct edges; 6. Stool without a defined shape; 7. Watery stool devoid of solid content.

### Statistical analysis

Statistical analysis was conducted using SPSS version 19.0 software. Data with a normal distribution were expressed as mean ± standard deviation (SD), while non-normally distributed data were presented as the median. Comparisons between groups were executed using the two-tailed Student’s t-test for normally distributed parameters. Additionally, statistical analysis was carried out using the R statistical framework. Significance was determined by a P-value of less than 0.05. Given the limited sample size, a power analysis was performed.

## Results

### Characteristics of children with FA

The 10 children diagnosed with FA exhibited distinct clinical symptoms, encompassing atopic eczema, diarrhea, cough, vomiting, angioneurotic edema, and more (refer to Table 1).

### Gut microbial Beta diversity in children

**Fig. 1:**
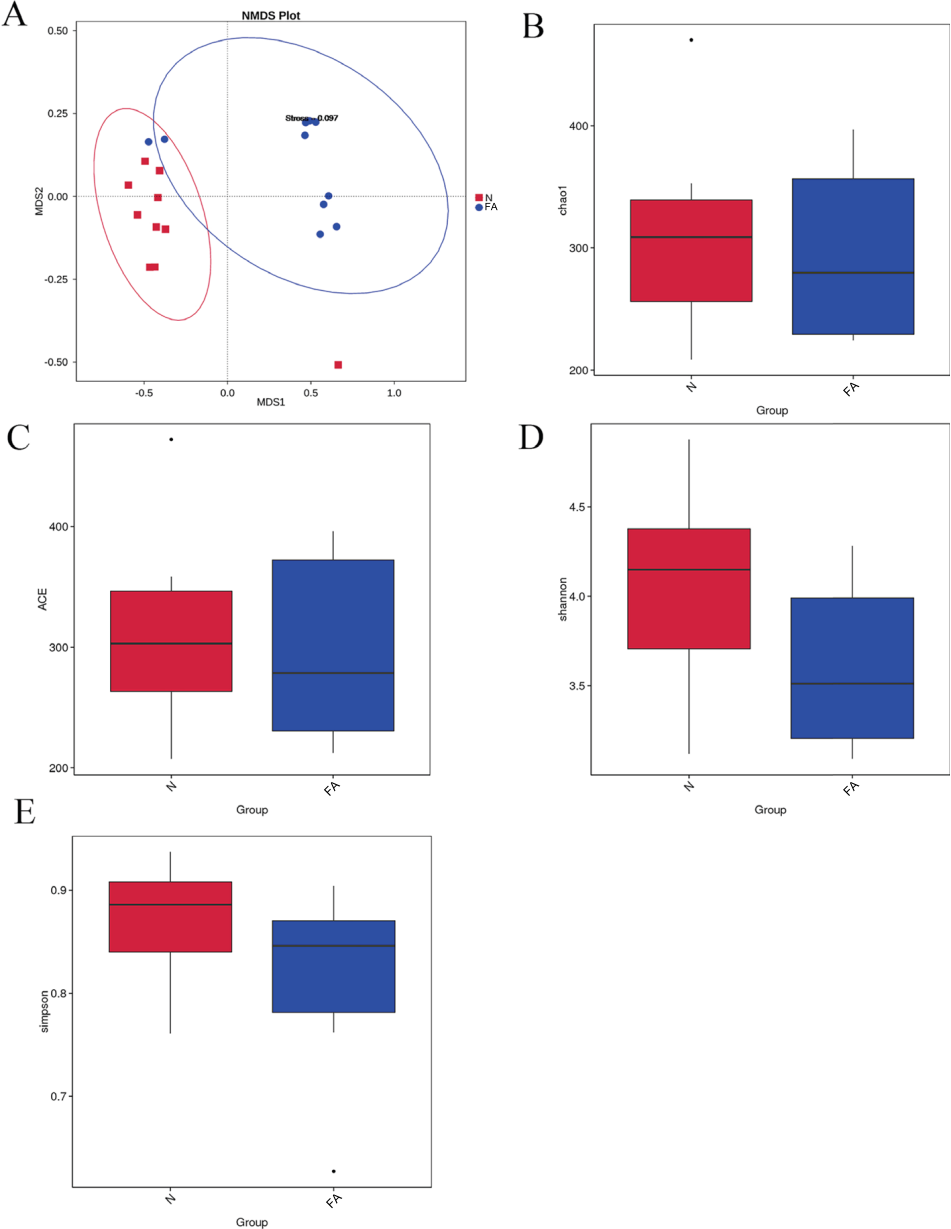
**A** The NMDS plot showed that the microbiota composition of FA and NC children were separated. **B** and **C** Species richness estimates: ACE and Chao1 indices. **D** and **E** Diversity estimates: Shannon and Simpson indices

In principal component analysis, a clear separation in microbiota composition between FA and non-FA children was observed (Fig. 1(A)). Evaluation of bacterial community characteristics, such as richness, diversity, and evenness, was performed using sequencing data, considering observed species count, Chao1, ACE, Shannon, and Simpson indices. Chao1 and ACE demonstrated comparable species richness between non-FA and FA children (Fig. 1(B) and (C)). In contrast, Shannon and Simpson indices indicated similar diversity and evenness of gut microbiota between FA children and non-FA children (Fig. 1(D) and (E)).

### Gut microbial composition in children

**Fig. 2:**
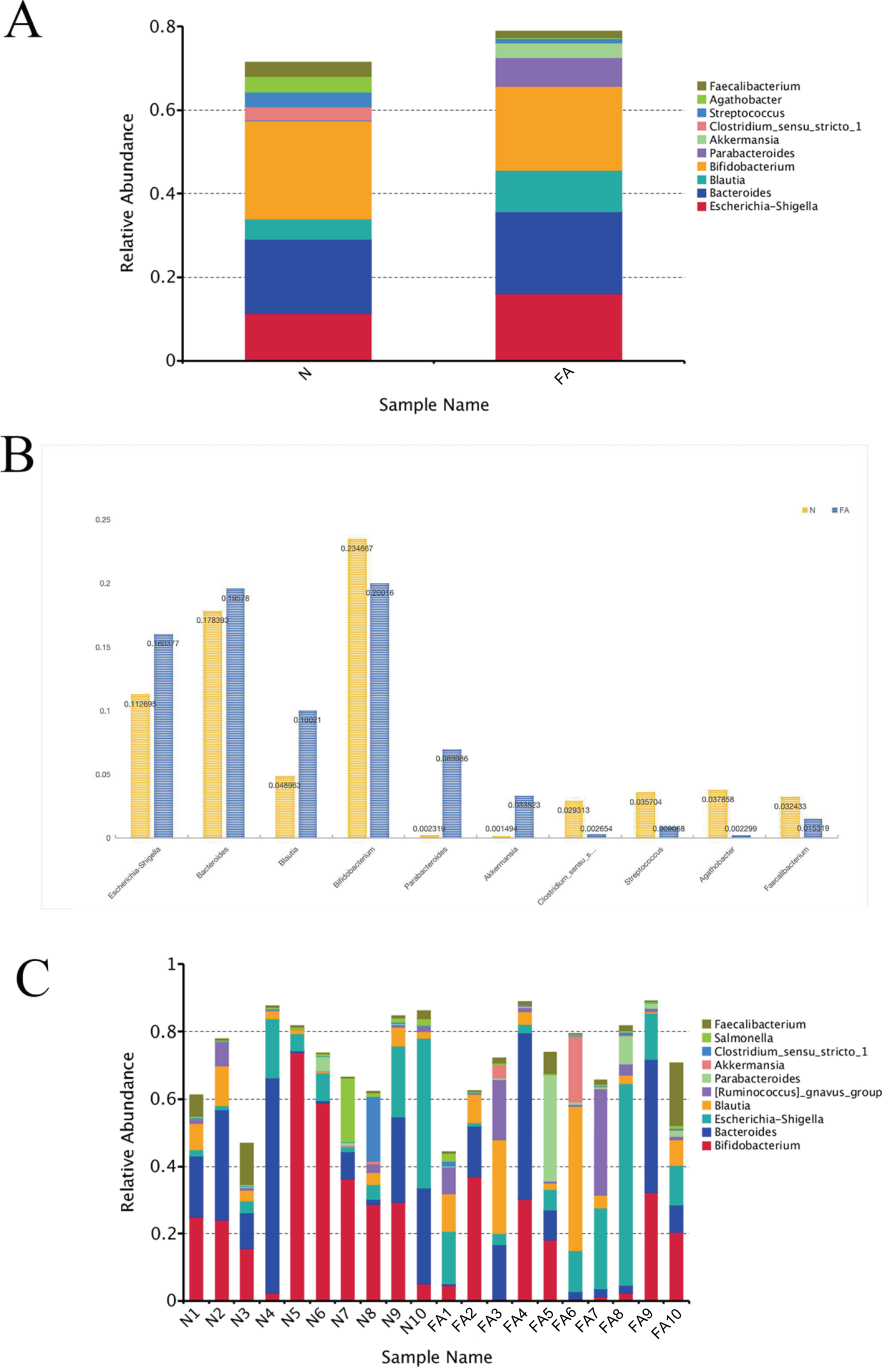
**A** Relative abundance of gut microbiota at genus level; **B** Comparison of the relative abundance of each bacteria at the level of genus between two groups; **C** The relative abundance of gut microbiota of individual samples in the children with food allergy group

The OTU dataset for FA and non-FA children was analyzed. At the genus level, FA children exhibited significantly higher relative abundances of *Akkermansia*, *Parabacteroides*, *Blautia*, and Escherichia-Shigella, while *Bifidobacterium* and *Clostridium* showed significantly lower relative abundances in FA children (Fig. 2(A) and (B)). On assessing individual FA children’s samples, we noted significant increases in *Escherichia-Shigella* in samples 7 and 8, accompanied by a noteworthy decrease in *Bifidobacterium* (Fig. 2(C)).

### Clinical manifestation of 10 children with food allergy after 2-year follow-up

At the conclusion of the 2-year period, all children reached the age of 3 years. Among the 8 FA children, either no clinical symptoms or mild symptoms were observed, allowing them to consume relevant allergenic foods. However, 2 patients (samples 7 and 8) continued to experience evident allergic symptoms. This observation led us to speculate that *Escherichia-Shigella* and *Bifidobacterium* could play substantial roles in the pathogenesis and development of FA. Importantly, after the 2-year span, the weight and height of the children remained largely unaffected (refer to Table 2).

**Table 2:** Clinical characteristics of the allergic diseases in 10 children after 2 years

Patient	Sex	Age(M)	Symptom	Treatment	Body weight (kg)	Height (cm)
1	F	48	Cough: Grade I	Leukotriene receptor antagonists	22.5	113
2	M	59	Eczema: scoard: 0 There was no recurrence of angioneurotic edema	Highly hydrolyzed protein formula was fed for 6 months, topical moisturizer	17.5	103
3	F	37	Stool grading: 4, atopic eczema: scoard: 0	Highly hydrolyzed protein formula was fed for 1 year, topical moisturizer	18	108
4	M	38	Stool grading: 4 atopic eczema: scoard: 0	No treatment was given	16	100
5	M	36	No vomiting, eczema: scoard:4.5%	Breast-feeding, topical moisturizer	17	100
6	M	38	Stool grading: 4	Highly hydrolyzed protein formula was fed for 6 months	16	104
7	M	42	Eczema: scoard: 40.5%, no dysphoria	Biologics and topical moisturizer	16	98
8	M	43	Eczema: scoard: 49.5%	Topical moisturizer and glucocorticoid	15	92
9	M	48	Eczema: scoard: 0 Stool grading: 4 No hypoproteinemia, no anemia	Amino acid formula feeding for half a year, highly hydrolyzed protein formula was fed for 1 year	20	110
10	F	60	Eczema:scoard: 4.5%, no dysphoria	Highly hydrolyzed protein formula was fed for 1 year	17.5	120

### Power analysis

The obtained results revealed that the power values across 10 tests ranged from 0.78 to 0.93, with an average power of 0.853. This study’s cumulative statistical power (determined through post-hoc power analysis) was deemed satisfactory at 85.3%. The post-hoc power analysis further confirmed that our tests fulfilled the power requirement of 0.8.

## Discussion

The gut microbiota has long been a focal point in the investigation of allergic diseases. Identifying potentially beneficial or harmful bacteria associated with FA holds immense potential for uncovering preventive measures and therapeutic targets. Despite reports comparing intestinal flora composition between healthy and FA-affected children, outcomes have remained contentious [12, 13]. This disparity might be attributed to factors such as subjects’ age, type of food hypersensitivity, dietary habits, and geographical location.

In our study, a post hoc power analysis indicated that our sample size was sufficient to meet the minimum requirement of achieving 80% power. Furthermore, the post hoc power calculation demonstrated that our study achieved an 80% power at a 5% alpha level. Our investigation involved an assessment of the diversity and community structure of gut microbiota in young infants with FA and their non-FA counterparts in Wenzhou. To achieve this, we employed 16S rRNA gene amplicon sequencing. Similar to findings in other studies [14–20], our research did not reveal significant differences in bacterial beta diversity between allergic and non-allergic children. These results suggest that the reduction in diversity is likely unrelated to allergic symptoms. Our findings unveiled that sensitized infants were discerned by higher abundances of *Akkermansia*, *Parabacteroides*, *Blautia*, and Escherichia-Shigella, as well as lower abundances of *Bifidobacterium* and *Clostridium*. Escherichia-Shigella, identified as an opportunistic pathogen, could cause severe infections, particularly in individuals with compromised immune systems. This may elucidate why severe symptoms like diarrhea and hypoproteinemia can manifest in certain infants with severe food hypersensitivity. Previous studies, including the one by Ling et al., observed elevated *Escherichia-Shigella* levels in FA infants [21]. Additionally, other researchers found heightened *Escherichia-Shigella* presence in infants with atopic dermatitis [22]. Collectively, these findings imply that increased *Escherichia-Shigella* could serve as an allergy marker, potentially elevating the risk of FA. Conversely, *Bifidobacterium* is a bacterial group renowned for its health-promoting potential. In our investigation, infants with FA exhibited low relative abundance of *Bifidobacterium*. This dearth might contribute to FA development. Numerous studies have attested to the advantageous effects of *Bifidobacterium* in preventing allergic diseases. These anti-allergic effects could arise from *Bifidobacterium*’s role in modulating the intestinal barrier and immune system. Notably, extracellular polysaccharides from short *Bifidobacterium* WBBR04 and *Bifidobacterium longum* WBLO01 bolstered intestinal barrier integrity by attaching to the small intestine lining, thereby effectively isolating allergens and thwarting FA [23]. *Bifidobacterium longum* was found to curtail IgE response and mast cell degranulation. Although sensitizing markers were unaffected, noteworthy increases were observed in the expressions of regulatory genes like Forkhead Box p3 (Foxp3), transforming growth factor-β (TGF-β), and interleukin (IL)-10 ileum genes, alongside elevated levels of the kynurenine metabolite [24]. Furthermore, feeding *Bifidobacterium longum* to FA-afflicted mice led to diminished IL-17 levels [25].

Few studies have explored the impact of gut microbiota composition on the long-term clinical outcomes of allergic diseases in early-life children with FA. Azad’s research [26] discovered that low species richness in the gut microbiota of 3-month-old children correlated with an elevated likelihood of food sensitization at 1 year of age. The Canadian Healthy Infant Longitudinal Development study reported that higher species richness at 3 months of age was linked to a 55% lower risk of FA at 1 year of age [27]. Furthermore, the study noted that a greater ratio of *Enterobacteriaceae*/*Bacteroidaceae* or elevated abundance of *Bacteroidaceae* species corresponded to a twofold increase in children’s risk of food sensitization. These investigations mainly focused on the risk of developing allergic diseases in otherwise healthy children later in life due to shifts in gut microbiota. Our study aimed to assess how gut microbiota composition influences the prognosis of allergic diseases in early-life children with FA. Among the 10 children we studied, 8 exhibited significant symptom relief after 2 years, while 2 continued to experience allergic symptoms. Notably, in these two cases, the early childhood intestinal flora displayed an increased presence of *Escherichia-Shigella* and a decreased level of *Bifidobacterium*. The gut microbiota composition in the remaining eight cases differed from those in samples 7 and 8. These eight cases did not show heightened *Escherichia-Shigella* and reduced *Bifidobacterium* levels. This suggests a potential link between the gut microbiota composition in early life, specifically the presence of *Escherichia-Shigella* and *Bifidobacterium*, and the long-term prognosis of FA in children. Addressing FA predominantly involves symptomatic management and the avoidance of allergenic foods. Children undergoing this process are at a critical stage of growth and development. Inadequate nutritional management during allergenic food avoidance can lead to growth delays and other problems, causing varying degrees of malnutrition. Different studies have yielded conflicting results regarding the impact of FA on growth. While some indicated growth impairment in FA infants and young children [28], others found no significant growth effects [29]. In our study, body growth parameters (weight and height) fell within the normal range according to World Health Organization (WHO) growth references. Our research delved into the impact of early-life gut microbiota dysbiosis on long-term clinical outcomes for children with FA. We discussed the prevalence of dominant and deficient bacteria in FA cases, offering a theoretical foundation for FA treatment. By modulating the dominant bacteria and addressing bacterial deficiencies, we can potentially devise therapeutic approaches to mitigate the enduring effects of FA on children. Nevertheless, our study has limitations, including a small sample size and a lack of fecal samples at the 2-year follow-up. This prevented us from establishing connections between early-life microbiota shifts and the resolution or persistence of allergic symptoms post-follow-up. Future research will involve enlarging the sample size, intensifying the monitoring of gut microbiota composition changes alongside corresponding clinical manifestations over a 2-year or longer span. The study will further explore the mechanisms underlying the long-term effects of gut microbiota on children with food allergies.

## Conclusion

In our study, it appears that the presence of FA and its subsequent prognosis might not be directly associated with the diversity of gut microbiota during infancy. However, the need for further confirmation through additional experiments in the future is evident, as the current study is limited by its small sample size.

## Data Availability

The datasets used and/or analysed during the current study are available from the corresponding author on reasonable request.

## Abbreviations

FA: Food allergy
OTUs: Operational taxonomic units
NMDS: Nonmetric multidimensional scaling
NC: Normal control
